# Regular Meeting Fulton County Medical Society, February 5, 1914

**Published:** 1914-02

**Authors:** R. R. Daly


### REGULAR MEETING FULTON COUNTY MEDICAL SOCIETY, FEBRUARY 5, 1914.

REPORTED BY R. R. DALY, M. D.

Dr. Dunbar Roy reported a case of unusual injury to the eye and exhibited the eye after removal with the foreign body still imbedded.

The boy was accidentally shot on the 21st of December, 1913. The leaden shot entered the face at the outer margin of the orbit, passed through the bone, entered the eye and seemed to be lodged in the nasal side of the body of the eye. After reaction, there was fair vision, but the case was not under observation at that time. On the 9th of January, vision began to fail, and on the 13th, the pupil was white, but with motile iris. The iris responded to light and was also so tremulous as with dislocated lense. An attempt was made to remove the shot so as to save the eye for cosmetic purposes and a skiagram was made by Dr. Derr. The shot was plainly to be seen in the radiograph, and measurements were taken to locate it exactly. Then an incision was made, but the body was not found.

Later on, it became necessary to enucleate and then it was discovered that the shot was imbedded in the sclera of the nasal side, but not at the point indicated by the X-ray. It had been nearly impossible to secure a perspective, owing to the final position of the shot. Of course, the electro-magnet was of no use because of the leaden character of the object. The shot had become encysted and could not have been removed, even if located. Had it been in any other part of the eye, the radiographs would have been of exact value.

Dr. E. G. Ballenger showed a case of Acne Vulgaris that had been very severe one time, but which was now 95 per cent well under the administration of frequently repeated doses of Salvarsan. There was absolutely no history of Syphilis.

Dr. Bunce asked if autogenous vaccine had been used, and if so, what organism had been found. He said that unless the acne bacillus were incorporated in the vaccine, it would be of no use. It was answered that this had been tried by other attending physicians, and it was not known as to the bacillus.

Dr. Cartlege said that he had treated several cases of acne recently with autogenous vaccine, properly constituted, and had had success. He had aided the treatment with the high frequency electric spray applied to the face.

Dr. R. M. Nelson said that he had seen many eye injuries in Panama, and that a notable one gave the history of having something strike him from a passing train. It caused little distress at the moment, and it could not be found. Shortly thereafter, there was complete degeneration of the optic nerve, and his conclusion is that a sharp foreign body penetrated the eye and buried itself in the nerve.

Dr. H. M. Lokey reported a case that perplexed him. A gentleman awakened in the Pullman car and found that he could not see with his left eye. Later in the morning, he could use it again. During the day, the blindness recurred several times, and the following day he sought advice. At that time the vision was 20/15 the blood pressure was 138, the urine showed Sp. gr. 1030, no albumin or sugar, but with much indican. The pupil was normal. An attack occurred a little later while he was in the office and the eye was seen. There was some haziness, the capillaries were swollen and the retina was very red. Vision returned in 25 minutes, and the redness disappeared. Lokey doesn't know what it is nor what to do.

Dr. Roy said that a German had reported a few such cases and had concluded that they were toxic. He advises rest and elimination.

Dr. Dorsey said that the heart should be carefully examined for infectious endocarditis. Embolic formation would account for all the symptoms mentioned.

Dr. Cartlege said that he recalled Dr. Calhoun's reporting similar cases to the Society some time ago, and these were autotoxic.

Dr. C. A. Rhodes read a paper, "A Free Clinic, and Does It Pay?" It appears elsewhere in the Journal.

Dr. Daly said, in discussing it, that Rhodes had answered the question asked, and there was no doubt but that the clinic in the Mill Settlement was paying from every point of view. He, himself, was familiar with the conditions, and had tried to cope with them several years ago, but had been unable to secure sufficient co-operation. As to clinics in general, they paid or not depending upon whether they reached the end set for them. If the gain was to be solely to the patient, they would be measured in that way. If the experience gained by the doctors or the students attached thereto were the criterion, another measurement was needed. If all-around improvement and general gain in sanitation and health were the object, then the ultimate results must be observed, and to do this the social service and "follow-up" work must be added to that of the doctors.

Dr. Cabot, of the Massachusetts General, had clearly shown the great waste that encumbered a clinic unless the follow-up was as carefully administered as any other part. In Daly's experience in his own clinic, where there were no students to educate, the follow-up was essential, and that part was done carefully every day.

Dr. Cecil Stockard said that his clinical experience was in the College, and that he had to measure the value to the doctor and students. The follow-up was necessarily very limited. He knew, of course, that the clinic paid as a teaching medium. Another point in connection with free clinics was the presence there of patients who were able to pay. He thought that a more extended investigation should be made as to their financial condition before admission so that injury would not be done to doctors in their private practise.

Dr. Rhodes said in closing that the follow-up idea was being incorporated into his work as rapidly as possible, and that there was no doubt of its value. As to investigation, all his patients were well known and there was no question of their indigency. A

public spirit was being aroused amongst them, and he had bright hopes of seeing a model village grow from the discordant units around the Mills.

Dr. A. B. Elkin read a paper on "Present-Day Faith in Sputum Examinations in Its Relation to Pulmonary Tuberculosis." It appears elsewhere in the Journal.

Dr. E. C. Thrash said he had worshipped at the shrines of all the gods who pretend to give a positive diagnosis in tuberculosis. He now has to go back to the careful findings of exhaustive examination of every individual case. Sputum is only of positive value when the germs are found, and there is grave danger in the patient's concluding he is well when the findings are negative, though the disease may be present.

Bronchiectasis throws the germs to the background. Large pus formations and purulent bronchorhea may hide the germs. These are not the important cases, because the patient is evidently sick, anyway, but where the germs are enclosed in cellular exudate and there are positive physical signs of the disease, there is danger of the patient over doing himself or of disseminating the trouble. Infrequently, as in syphilis and cancer, we may have positive physical signs for a time and not the disease in question. But these soon clear the situation for themselves.

Dr. R. T. Dorsey said that the diagnosis of tuberculosis depended upon the physical signs, clinical history and the bacilli in the sputum. All others, such as tuberculin tests, cyto-diagnosis, inoculation of guinea pigs, radiographs, etc., are not reliable.

He exercised special care in the selection of the specimens of sputum he wished to examine. He gives potas iodid for a short time to increase the secretions, and then dries them after precipitation. This concentration will frequently enable him to find germs where he has failed in the usual method.

Dr. C. W. Gould said that in military hospitals where the discipline was greater, the patients could be regularly examined many times under controlled conditions and often a case would be negative for several days and then show the germ. He advocates the laboratory method in conjunction with the others, because nothing may be neglected that would aid in controlling this disease.

Dr. Nelson related Dr. Brennin's experience in Panama. It seems that they were finding an unusual number of positive cases in sputum tests that did not correspond with the clinical history. They discovered that the water being used contained bacilli that were acid-fast. As soon as distilled water was resorted to, these cases disappeared.

Dr. Kendrick said that for 25 years the tubercular poor came to his clinic, but of late they have gone to the care of the Anti-Tuberculosis Association. The microscope is of value, of course, but the incipient cases can be determined in four-fifths of the instances without it. The temperature is the great guide, and if carefully watched will decide what the other signs mean.

Dr. Bunce said that he had used the anti-formine method of examining sputum for over a year and found it successful in 33 per cent of the specimens that were minus by the usual method.

Dr. J. L. Campbell said that he had used a method of his own for many years in examining Dr. Kendrick's cases. It was similar to the anti-formine method.

Dr. Elkin closed by saying that he had no idea of attacking the microscope, but he was speaking of the early cases.

